# Presentation of Coping Strategies Associated with Physical and Mental Health During Health Check-ups

**DOI:** 10.1007/s10597-016-0048-9

**Published:** 2016-08-11

**Authors:** Miho Ito, Eisuke Matsushima

**Affiliations:** 1 Section of Liaison Psychiatry and Palliative Medicine, Graduate School of Medical and Dental Sciences, Tokyo Medical and Dental University, 1-5-45 Yushima, Bunkyo-ku, Tokyo, 113-8519 Japan; 2 Koganehara Medical Examination Clinic, Seikokai Medical Corporation, 3 Kogane, Matsudo, Chiba 270-0014 Japan

**Keywords:** Coping, Depression screening, Cognitive behavioral therapy, Psychological approach, Primary care, Health check-up

## Abstract

We identified coping behaviors during regular health check-ups and examined whether they were related to physical and mental health. We assessed coping strategies with the Brief COPE scale in 201 people who underwent a regular health check-up in a clinic. We found several significant relationships between coping and physical/psychological conditions presented in health check-up: Humor and systolic blood pressure, Substance use and high-density lipoprotein cholesterol, Venting and low-density lipoprotein cholesterol, Self-blame and depression, and Behavioral disengagement and sleep disorder. By evaluating coping strategies and screening depression as part of a regular health check-up, General practitioner can provide psychological intervention such as cognitive behavioral therapy which may improve both mental and physical health of the people.

## Background

Regular health check-up and health guidance aim to help consumers to become aware of their health problems and improve their overall health. Health guidance mainly focuses on physical examination data and providing general advice on diet, physical activity and exercise, and other lifestyle habits. This method tends to produce only short-term effects. To improve overall outcomes, psychological approach improving individual skills for self-management and changing behavior should be considered. In the present study, we focused on coping behaviors and mental health of participants receiving regular health check-up. Coping is defined as the thoughts and behaviors used to manage the internal and external demands of stressful situations. It is not only an explanatory concept regarding the variability in response to a stressor but also a portal for cognitive-behavioral interventions (Folkman and Moskowitz 2004). Previous studies on coping have targeted patients, workers, medical staff, and students. To our knowledge, Hosaka et al. (1998, 2004) were the first to link coping to health check-ups. They introduced the “Stress-coping test” into a health check-up program and identified some relationships between coping behaviors and biochemical indices of the examinees. The measure that they created consists of four subscales: “active behavioral coping”, “distraction”, “passive resignation”, and “denial” (Hosaka et al. 1998) or “avoidance” (Hosaka et al. 2004). They recognized “active behavioral coping” and balanced “distraction” as important coping skills from the perspective of preventive medicine. However, their study had two limitations. First, the total variances of coping variables accounting for biomedical indices were very small. Second, the subscales of the measure were simple. Another measure should therefore be considered to explain additional variance of coping. We used a shortened version of the COPE Inventory (Carver et al. 1989), which is the most frequently used measure to assess individual coping strategies in the English literature, called the Brief COPE (Carver 1997). We hypothesized that coping behaviors would predict physical and mental health. We examined the link between coping behaviors and physical/psychological conditions presented in health check-ups to provide refined health instruction based on individual coping style.

## Methods

### Participants

Participants were recruited by consecutive sampling from 209 examinees that had an annual health check-up at the Koganehara Medical Examination Clinic (Chiba, Japan) between July and December 2013. Participants who met the following criteria were eligible: (1) 20 years or older, (2) undergoing a comprehensive check-up including biochemical tests, (3) no apparent history of psychiatric disorders, and (4) no critical problems found during the current check-up. Participants who did not undergo biochemical tests (n = 4), who refused to attend (n = 3), and who were being treated for schizophrenia (n = 1) were excluded. Participants taking medications for hypertension or hyperlipidemia were included. A total of 201 participants remained (118 males, 83 females; mean age ± standard deviation, 51.24 ± 1.00 years; range, 23–74 years).

## Measures

The check-up program included recording the medical history, a physical examination, and laboratory tests. We used body mass index (BMI), blood pressure, triglycerides (TG), high-density lipoprotein cholesterol (HDL), low-density lipoprotein cholesterol (LDL), and fasting blood glucose (FBG) from standard items for health check-ups as measures of physical condition. Participants filled out an interview questionnaire about their personal and family history of disease, medication, smoking status, and alcohol consumption. Blood pressure was measured with a mercury manometer with the examinee in a sitting position. Weight and height were measured with an auto-scale, and BMI was calculated. Serum samples were collected in a fasting state. Participants were provided with questionnaires including the Brief COPE (Carver 1997), the Athens Insomnia Scale (AIS) (Soldatos et al. 2000), and the two-question case-finding instrument (TQ) (Whooley et al. 1997). The items included on the Brief COPE measure 14 coping concepts. Every coping scale consists of two items and is rated using a four-point Likert-type scale that ranges from “I usually don’t do this at all (score 1)” to “I usually do this a lot (score 4)”. The 14 concepts are Self-distraction, Active coping, Denial, Substance use, Use of emotional support, Use of instrumental support, Behavioral disengagement, Venting, Positive reframing, Planning, Humor, Acceptance, Religion, and Self-blame. The Japanese version of the Brief COPE (Otsuka et al. 2009) was developed by Carver, with the method of forward and backward translation by two bilingual speakers. The AIS (Soldatos et al. 2000) is a self-assessment psychometric instrument that was designed to quantify sleep difficulty based on the International Classification of Diseases, 10th edition (ICD-10) (World Health Organization 1992). The AIS can be utilized in clinical practice and research, not only as an instrument to measure the intensity of sleep-related problems but also as a reliable screening tool for the diagnosis of insomnia (Soldatos et al. 2003). It consists of eight items: difficulty with sleep induction, awakening during the night, early morning awakening, total sleep time, overall quality of sleep, problems with sense of well-being, functioning, and sleepiness during the day. Every item is rated from “no problem at all (score 0)” to “a very serious problem (score 3)”. The total score of the AIS is interpreted as follows: a score of 0 to 3 means “no concern about a sleep disorder”, a score of 4 or 5 means “sleep disorder is slightly suspected”, and a score of 6 or higher means “sleep disorder is suspected” (Soldatos et al. 2000). TQ is a useful measure for the detection of depression. The first question is “During the past month, have you often been bothered by feeling down, depressed, or hopeless?” and the second question is “During the past month, have you often been bothered by little interest or pleasure in doing things?” A negative response to both questions made depression highly unlikely. For people who answer yes to either of the questions, a diagnosis of depression can be confirmed in the presence of other related symptoms such as fatigue, restlessness, guilt, poor concentration, suicidal ideation, and change in sleep or appetite (Whooley et al. 1997). In the current study, we defined those who responded “yes” to either of the two questions as depressed and people who responded “no” to both questions as non-depressed.

## Procedure

The protocol for this study was approved by the institutional ethics committee of Tokyo Medical and Dental University (approval number 1498). After their health check-ups, the participants were briefed on the details of this study and read an informed consent form stating that the participation was voluntary and confidential and that they had the right to withdraw from this study at any time. Participants were provided with self-administered questionnaires during their clinic visit. Those questionnaires were collected during the visit and numbered for tracking purposes without any names to ensure confidentiality.

### Statistical Analyses

Each scale of the Brief COPE was analyzed by demographic and clinical variables. We used Student’s *t* tests to compare sex, age and depression status. We conducted a one-way analysis of variance (ANOVA) to compare the total AIS score, followed by Tukey’s test for multiple comparisons. Other appropriate non-parametric tests were used when distributional assumptions were in doubt. We examined how coping variables related to physical condition by hierarchical multiple regression analyses for blood pressure, TG, HDL, LDL and FBG. In step 1, age, male sex, smoking, medication, and laboratory tests were set. In step 2, coping scales were entered. Sex, smoking and medication status were dummy-coded for use in the regression analyses: 1 = male, 0 = female; 1 = smoker, 0 = non-smoker; 1 = medicated for hypertension or hyperlipidemia, 0 = non-medicated. A p value <0.05 was considered statistically significant for all analyses. All data were analyzed using Statcel 3 by OMS Publishing (Tokyo, Japan).

## Results

Of the 201 participants, 35.3 % (n = 71) were suspected to be depressive, and 18.9 % (n = 38) were suspected to have a sleep disorder. Individuals who had omissions on the Brief COPE (planning, n = 3; Humor, n = 1; Behavioral disengagement, n = 1; and Acceptance, n = 1) or the AIS (n = 2) were excluded from the corresponding analyses.

Table 1 shows the coping scores of each sub-scale compared by sex, age, depressive status, and total AIS score. Women scored significantly higher than men in Self-distraction, Active coping, Use of emotional support and Use of instrumental support. Men scored significantly higher than women in Substance use. We divided participants into two groups according to age: the young/middle-aged group (23–59 years; n = 149) and the old-aged group (60–74 years; n = 52). The young/middle-aged group scored significantly higher in Use of emotional support, Use of instrumental support and Humor than the old-aged group. The old-aged group scored significantly higher in Denial than the young/middle-aged group. Depressed people scored significantly higher in Self-blame than non-depressed people. We divided participants into three groups by total AIS score. The group of people with a total AIS score of 6 or higher scored higher in Behavioral disengagement than the group with a total AIS score of 0 to 3.

**Table 1:** Scores of each coping scale of the Brief COPE compared by sex, age, depressive status, and total AIS score

Coping scales	Overall n = 201	Sex			Age			Depressive status			Overall n = 199	AIS total score			
		Male n = 118	Female n = 83	p	Young/middle (23–59 years) n = 149	Old (60–74 years) n = 52	p	Non-depressed n = 130	Depressed n = 71	p		0–3 n = 118	4–5 n = 43	≥6 n = 38	p
		Mean (SD)			Mean (SD)			Mean (SD)				Mean (SD)			
Self-distraction		**4.94 (1.14)**	**5.36 (1.08)**	0.01*	5.19 (1.18)	4.88 (0.94)	0.09	5.10 (1.06)	5.14 (1.26)	0.81		5.14 (1.21)	5.05 (1.02)	5.08 (1.05)	0.88
Active		**5.70 (0.96)**	**6.06 (0.97)**	0.01*	5.89 (1.00)	5.75 (0.93)	0.39	5.88 (0.92)	5.80 (1.09)	0.61		5.93 (0.93)	5.65 (1.00)	5.84 (1.10)	0.27
Denial		3.37 (1.27)	3.42 (1.11)	0.78	**3.19 (1.14)**	**3.98 (1.23)**	<0.01*	3.43 (1.16)	3.32 (1.30)	0.55		3.44 (1.28)	3.30 (1.12)	3.42 (1.08)	0.81
Substance use		**3.69 (1.65)**	**3.05 (1.44)**	<0.01*	3.46 (1.52)	3.31 (1.79)	0.55	3.36 (1.58)	3.54 (1.63)	0.46		3.31 (1.54)	3.37 (1.51)	3.92 (1.78)	0.11
Emotional support		**4.33 (1.07)**	**4.89 (1.27)**	<0.01*	**4.71 (1.20)**	**4.13 (1.05)**	<0.01*	4.48 (1.06)	4.70 (1.39)	0.25		4.44 (1.12)	4.58 (1.01)	4.89 (1.52)	0.12
Instrumental support		**4.72 (1.11)**	**5.07 (1.34)**	0.04*	**5.00 (1.21)**	**4.48 (1.16)**	0.01*	4.82 (1.08)	4.96 (1.44)	0.47		4.83 (1.20)	4.74 (1.03)	5.13 (1.47)	0.31
Behavioral disengagement	(n = 200)	4.03 (1.06)	4.01 (0.94)	0.88	4.03 (1.02)	4.00 (0.98)	0.84	3.91 (0.81)	4.23 (1.28)	0.07	(n = 198)	**3.88 (0.89)**	4.21 (1.01)	**4.34 (1.21)**	0.02*
Venting		4.57 (1.04)	4.83 (1.17)	0.09	4.76 (1.14)	4.44 (0.96)	0.07	4.64 (1.06)	4.75 (1.18)	0.51		4.66 (1.13)	4.63 (1.00)	4.82 (1.14)	0.71
Positive reframing		5.44 (1.06)	5.72 (1.11)	0.07	5.54 (1.09)	5.60 (1.12)	0.77	5.61 (1.07)	5.46 (1.13)	0.38		5.63 (0.99)	5.53 (1.20)	5.37 (1.28)	0.44
Planning	(n = 198)	5.68 (1.15)	5.88 (1.32)	0.28	5.80 (1.20)	5.67 (1.29)	0.52	5.76 (1.14)	5.76 (1.36)	0.99	(n = 196)	5.79 (1.16)	5.76 (1.28)	5.62 (1.36)	0.76
Humor	(n = 200)	4.31 (1.20)	4.21 (1.21)	0.54	**4.41 (1.23)**	**3.87 (1.02)**	0.01*	4.37 (1.22)	4.08 (1.16)	0.11	(n = 198)	4.15 (1.15)	4.72 (1.44)	4.18 (0.98)	0.05
Acceptance	(n = 200)	5.86 (1.02)	5.99 (0.99)	0.36	5.99 (1.00)	5.69 (1.00)	0.06	5.88 (1.03)	5.96 (0.96)	0.62	(n = 198)	5.91 (0.97)	6.05 (1.07)	5.76 (1.05)	0.45
Religion		3.17 (1.30)	3.53 (1.54)	0.07	3.23 (1.43)	3.58 (1.33)	0.12	3.28 (132)	3.38 (1.56)	0.65		3.19 (1.40)	3.53 (1.32)	3.55 (1.54)	0.22
Self-blame		4.46 (1.14)	4.70 (1.23)	0.16	4.49 (1.22)	4.75 (1.06)	0.17	**4.40 (1.12)**	**4.85 (1.24)**	0.01*		4.42 (1.19)	4.60 (1.11)	4.95 (1.14)	0.05

*AIS* athens insomnia scale

P values were calculated by student *t* test for sex, age, and depressive status

P values were calculated by one-way ANOVA for sleep quality

Non-parametric tests were used when distributional assumptions were in doubt

The results that are statistically significant are typed in bold

Asterisk indicates statistically significance with p-value < 0.05

Age, male sex, and BMI were significantly related to systolic blood pressure in step 1. In step 2, age, male sex and Humor were significantly related to higher systolic blood pressure (Table 2).

**Table 2:** Hierarchical multiple regression analysis for variables predicting systolic blood pressure

Variables	Step 1				Step 2			
	B	SE of B	β	p	B	SE of B	β	p
Age	0.22	0.11	0.15	0.04*	0.32	0.12	0.23	0.01*
Male sex	5.33	2.43	0.17	0.03*	7.13	2.65	0.23	0.01*
Smoking	−3.64	2.42	−0.10	0.13	−4.08	2.45	−0.12	0.10
Antihypertensive agents	5.04	2.93	0.12	0.09	5.61	3.10	0.14	0.07
BMI	0.82	0.37	0.18	0.03*	0.50	0.38	0.11	0.19
TG	−0.01	0.02	−0.04	0.64	−0.01	0.02	−0.03	0.71
LDL/HDL	−6.60	10.15	−0.06	0.52	−8.51	10.23	−0.07	0.41
FBG	0.11	0.06	0.13	0.07	0.06	0.06	0.06	0.36
Self-distraction					1.94	1.04	0.14	0.06
Active					0.28	1.36	0.02	0.84
Denial					0.51	1.08	0.04	0.63
Substance use					0.41	0.74	0.04	0.58
Emotional support					−2.15	1.33	−0.17	0.11
Instrumental support					2.14	1.30	0.17	0.10
Behavioral disengagement					0.19	1.25	0.01	0.88
Venting					1.41	1.04	0.10	0.18
Positive reframing					−1.02	1.21	−0.07	0.40
Planning					−0.69	1.19	−0.06	0.56
Humor					2.68	0.98	0.21	0.01*
Acceptance					1.10	1.29	0.07	0.39
Religion					0.47	0.80	0.04	0.56
Self-blame					−0.67	0.93	−0.05	0.47
Adjusted R^2^		0.18				0.28		

*HDL* high-density lipoprotein cholesterol, *R* ^*2*^ coefficient of correlation, *B* unstandardized regression coefficient, *SE* standard error, *β* standardized partial regression coefficient, *BMI* body mass index, *TG*, triglycerides, *LDL* low-density lipoprotein cholesterol, *HDL* high-density lipoprotein cholesterol, *FBG* fasting blood glucose

Asterisk indicates statistically significance with p-value < 0.05

Age, male sex, BMI, triglyceride level and Substance use were all significantly related to HDL in the final step (Table 3). Age, smoking, use of antihyperlipidemic agents, diastolic blood pressure and Venting were significantly related to LDL in the final step (Table 4). No coping scale was significantly related to diastolic blood pressure, TG, or FBG.

**Table 3:** Hierarchical multiple regression analysis for variables predicting HDL

Variables	Step 1				Step 2			
	B	SE of B	β	p	B	SE of B	β	p
Age	0.21	0.09	0.15	0.01*	0.20	0.10	0.14	0.04*
Male sex	−11.71	2.12	−0.38	<0.01*	−12.24	2.28	−0.39	<0.01*
Smoking	1.41	2.06	0.04	0.49	1.05	2.08	0.03	0.61
Antihyperlipidemic agents	−3.22	3.04	−0.06	0.29	−3.44	3.03	−0.07	0.26
BMI	−0.98	0.31	−0.21	<0.01*	−1.06	0.32	−0.23	<0.01*
SBP	−0.01	0.08	−0.01	0.94	−0.05	0.09	−0.05	0.58
DBP	0.19	0.13	0.13	0.13	0.22	0.13	0.15	0.10
TG	−0.05	0.01	−0.26	<0.01*	−0.04	0.01	−0.24	<0.01*
LDL	−0.06	0.03	−0.11	0.07	−0.06	0.03	−0.11	0.08
FBG	0.00	0.05	0.00	0.95	0.00	0.05	0.00	0.98
Self-distraction					0.39	0.88	0.03	0.66
Active					−0.64	1.14	−0.04	0.57
Denial					0.39	0.91	0.03	0.67
Substance use					1.83	0.61	0.19	<0.01*
Emotional support					0.30	1.12	0.02	0.79
Instrumental support					−0.49	1.11	−0.04	0.65
Behavioral disengagement					−1.68	1.03	−0.11	0.11
Venting					1.35	0.89	0.10	0.13
Positive reframing					1.45	1.01	0.10	0.16
Planning					−1.54	0.99	−0.12	0.12
Humor					−0.34	0.85	−0.03	0.68
Acceptance					0.86	1.07	0.06	0.42
Religion					−0.11	0.68	−0.01	0.87
Self-blame					−0.39	0.79	−0.03	0.62
Adjusted R^2^		0.42				0.49		

*HDL* high-density lipoprotein cholesterol, *R* ^*2*^ coefficient of correlation, *TG* triglycerides, *LDL* low-density lipoprotein cholesterol, *FBG* fasting blood glucose, *B* unstandardized regression coefficient, *SE* standard error, *β* standardized partial regression coefficient, *BMI* body mass index, *SBP* systolic blood pressure, *DBP* diastolic blood pressure

Asterisk indicates statistically significance with p-value < 0.05

**Table 4:** Hierarchical multiple regression analysis for variables predicting LDL-cholesterol

Variables	Step 1				Step 2			
	B	SE of B	β	p	B	SE of B	β	p
Age	0.62	0.20	0.23	<0.01*	0.63	0.23	0.23	<0.01*
Male sex	−9.32	5.19	−0.16	0.07	−7.75	5.82	−0.13	0.18
Smoking	9.74	4.67	0.15	0.04*	10.61	4.89	0.16	0.03*
Antihyperlipidemic agents	−16.90	6.89	−0.17	0.02*	−17.42	7.13	−0.18	0.02*
BMI	1.35	0.73	0.15	0.07	1.58	0.78	0.18	0.05
SBP	−0.36	0.19	−0.19	0.06	−0.34	0.21	−0.18	0.11
DBP	0.64	0.29	0.23	0.03*	0.62	0.31	0.23	0.04*
TG	−0.02	0.03	−0.05	0.52	−0.01	0.03	−0.04	0.61
HDL	−0.30	0.17	−0.16	0.07	−0.31	0.18	−0.17	0.08
FBG	−0.20	0.12	−0.12	0.10	−0.20	0.12	−0.12	0.11
Self-distraction					−1.74	2.09	−0.07	0.41
Active					−0.09	2.71	0.00	0.97
Denial					0.88	2.16	0.04	0.69
Substance use					−1.85	1.48	−0.10	0.21
Emotional support					2.17	2.67	0.09	0.41
Instrumental support					−1.25	2.63	−0.05	0.64
Behavioral disengagement					−1.12	2.47	−0.04	0.65
Venting					4.79	2.10	0.18	0.02*
Positive reframing					1.72	2.42	0.06	0.48
Planning					−1.91	2.36	−0.08	0.42
Humor					−0.82	2.01	−0.03	0.69
Acceptance					−0.80	2.56	−0.03	0.75
Religion					−0.07	1.61	0.00	0.96
Self-blame					0.60	1.87	0.02	0.75
Adjusted R^2^		0.14				0.18		

*LDL* low-density lipoprotein cholesterol, *R* ^*2*^ coefficient of correlation, *B* unstandardized regression coefficient, *SE* standard error, *β* standardized partial regression coefficient, *BMI* body mass index, *SBP* systolic blood pressure, *DBP* diastolic blood pressure, *TG* triglycerides, *HDL* high-density lipoprotein cholesterol, *FBG* fasting blood glucose

Asterisk indicates statistically significance with p-value < 0.05

## Discussion

Depression and anxiety disorders are highly prevalent disorders (Høifødt et al. 2011). In the current study, more than 35 % of the participants were suspected to be depressive. General practitioner is often the first to evaluate people with depression. TQ has been utilized for depression screening: A positive response to the two-item instrument had a sensitivity of 96 % (95 % confidence interval 90–99 %) and a specificity of 57 % (95 % confidence interval 53–62 %) (Whooley et al. 1997). Ultimate determination of depression must be done carefully and correctly, however, TQ can help General practitioner to identify people who may need to consult psychiatrists and psychologists.

Our results demonstrated sex differences in coping: women were more likely to use Self-distraction, Active coping, Emotional support, and Instrumental support, whereas men were more likely to exhibit Substance use. Our results are in line with those of previous studies that indicated that females were more likely to use Positive coping (Cherkil et al. 2013) and Support coping (Cherkil et al. 2013; Folkman et al. 1987; Hanninen and Aro 1996; Horwitz et al. 2011; Karekla and Panayiotou 2011; Tomotsune et al. 2009), whereas males preferred Substance Use (Cherkil et al. 2013). In this context, sex differences in coping should be considered regarding health instruction for examinees. Attention to alcohol consumption is more important for men. For women, Emotional or Instrumental support from a healthcare provider, such as counseling, information and advice, can be effective approaches. Furthermore, promoting Active coping and Self-distraction may increase good outcomes. Regardless of sex, Active coping and Self-distraction were both considered important (Hosaka et al. 1998, 2004).

Younger people scored higher for Use of emotional support, Use of instrumental support, and Humor, whereas older people scored higher for Denial. Except for Humor, our results were mostly in agreement with previous studies, which suggested that younger people used more active, interpersonal, problem-focused coping whereas older people used more passive, emotion-focused coping (Cherkil et al. 2013; Tomotsune et al. 2009). The coping styles undergo genetic and environmental changes with increasing age (Tomotsune et al. 2009), and the role of environmental influences becomes increasingly important during adulthood (Hur et al. 2012). Folkman and Lazarus (1980) suggested that older people who experience health-related problems favor emotion-focused coping, whereas younger people enduring family and work problems favor problem-focused coping mechanisms. The age-related differences in coping appear to be a function of the different types of stress associated with aging rather than age itself (Folkman and Lazarus 1980).

In line with previously reported literature (Brown et al. 2007; Fear et al. 2009; Hanninen and Aro 1996; Nielsen and Knardahl 2014; Otsuka et al. 2009; Stevenson et al. 2012), Self-blame, which is an emotion-focused coping mechanism, was significantly related to depression states in the current study. Coping strategies can have major impacts on life (Raheel 2014). For example, problem-focused coping strategies have decreased depression among adolescents, as well as later on in adulthood (Compas et al. 2001; Herman and Tetrick 2009). Emotion-focused coping strategies have been associated with mental health problems such as anxiety and depression (Suldo et al. 2008; Tolan et al. 2002). Adolescents need to be trained in overcoming maladaptive strategies to avoid depression and suicidal tendencies in the future (Raheel 2014). People, who resort to emotion-focused coping, as presented in health check-ups, should also be carefully observed. In the current study, Behavioral disengagement was significantly related to sleep disorders. Several studies have examined coping in regard to insomnia (LeBlanc et al. 2009; Morin et al. 2013; Pillai et al. 2014). Insomniacs have perceived their lives to be relatively more stressful than non-insomniacs, and the former relied more on maladaptive coping strategies than good sleepers (Morin et al. 2013). Coping skills were not associated with a new onset of insomnia (LeBlanc et al. 2009). Pillai et al. (2014) first examined intrusion as a prospective risk factor for insomnia, and found three specific coping factors were predictors of insomnia: Behavioral disengagement, Substance use and Self-distraction. Furthermore, they assessed whether these coping behaviors mediated the relationship between stress exposure and the development of insomnia and found that the three coping behaviors also acted as a mediator.

Blood pressure reactions depend on coping behavior (Sherwood et al. 1990), and the stress reactivity of hemodynamic parameters can be determined by structural and functional aspects of arterial vessels and baroreflex sensitivity (Lipman et al. 2002). High levels of self-efficacy in problem-focused coping were associated with a decrease in mean arterial pressure, systolic blood pressure, and pulse pressure (Harmell et al. 2011). Tasks that demand active coping strategies increased cardiac output and decreased vascular resistance, which resulted in an increase in blood pressure (Sherwood et al. 1990). Similarly, a high emotion-oriented coping score was associated with an increased risk of hypertension (Ariff et al. 2011). In the current study, Humor was one of predictors of systolic blood pressure. Humor is an emotion-oriented coping mechanism that is used to regulate emotional distress for unchangeable problematic situations (Celso et al. 2003). Humor has the effect of modifying (Fry 1994) and reappraising (McCrae 1984) stressors. Humor is also used to avoid thinking about stressors (McCrae 1984). The Inhibiting or distancing defense mechanism is one of the major characteristics of hypertensive patients (Sommers and Greenberg 1989).

In the current study, there were significant associations between Substance use and HDL and between Venting and LDL. TG had no significant association with any coping scale. Hosaka et al. (1998, 2004) examined the association between coping and blood lipid levels among health check-up examinees using their “Stress-coping test” and found a positive relationship between “distraction” and HDL. Considering that Substance use on the Brief COPE almost corresponds to “distraction” on the “Stress-coping test”, our results were consistent with their findings. Further, alcohol consumption has been known to increase HDL (Ellison et al. 2004). Based on these findings, we suggest that Substance use is a predictor of relatively high HDL. Martin et al. (2013) conducted a principle component analysis of the 28 items of the Brief COPE and examined the relationships among coping, lifestyle behaviors and cardiovascular risks in a non-medical sample of university students. In their analyses, the use of Social supportive coping, which included Venting, Emotional support and Instrumental support, was a significant predictor of calculated LDL. Our finding of a relationship between Venting and LDL partially agrees with that study. Venting refers to stating one’s unpleasant feelings or expressing one’s negative feelings (Carver 1997). This coping strategy is similar to Explosiveness of speech, one of the characteristics of the Type A behavior pattern. The Type A behavior pattern is another response to a stressor, which is characterized by extreme hostility, competitiveness, hurry, impatience, restlessness, aggressiveness, and explosiveness of speech. The Type A behavior pattern has been associated with an increased risk for coronary heart disease (Friedman and Rosenman 1959); however, the relationship with serum cholesterol has been often weak and inconsistent (Edwards and Baglioni 1991). Doornen and Orlebeke (1982) indicated that psychological stressors significantly elevated serum cholesterol concentration and psychological characteristics, such as Type A behavior patterns, were positively correlated with serum cholesterol concentration. However, a specific effect of Venting on LDL was inconclusive in the current study.

People with depression or anxiety prefer being treated in primary care setting for its accessibility, affordability, and less stigma (Høifødt et al. 2011). The most widely researched and used psychological approach in primary care is cognitive behavioral therapy (CBT) (Khoury and Ammar 2014). CBT teaches stress management techniques to cope with stressful situations more effectively. CBT attempts to change dysfunctional patterns of thinking and non-adaptive behaviors to prevent development and maintenance of symptoms of depression or anxiety (Beck 2011; Høifødt et al. 2011). CBT-based self-help with clinician support delivered in everyday primary care setting represents an effective treatment for depression and anxiety disorders. Such interventions supported by General practitioner, nurses, social workers or individuals with academic degrees in the mental health or health behavior field are effective (Høifødt et al. 2011). Making an effort to change maladaptive behaviors may also improve physical health associated with coping behaviors.

From these contexts, evaluating coping and screening depression as part of a regular health check-up is reasonable for primary care. With CBT based on such information, General practitioner can make psychological approach to health improvement of the people.

## Conclusion

To our knowledge, this is the first study assessing health check-up results related to coping behaviors with the Brief COPE. We found several significant relationships between coping and the physical/psychological conditions presented in health check-up. Evaluating coping strategies and screening depression as part of a regular health check-up is useful and worthwhile in primary care setting. General practitioner can approach psychologically with CBT which may improve both mental and physical health of the people.

The present study has several limitations. First, the sample size was fairly small. Second, the study period was only 6 months, and we did not observe the participants over an extended period of time. Third, we could not demonstrate a cause and effect relationship between coping behaviors and physical examination results. Further research is required to explain how coping behaviors affects physical and mental health in more detail.
